# Factors Associated With Neurosyphilis in Patients With Syphilis Treatment Failure: A Retrospective Study of 165 HIV-Negative Patients

**DOI:** 10.3389/fmed.2022.757354

**Published:** 2022-05-20

**Authors:** Wenying Cui, Junling Yan, Wenjia Weng, Yanqing Gao, Wei Zhu

**Affiliations:** ^1^Department of Dermatology, Xuanwu Hospital, Capital Medical University, Beijing, China; ^2^Department of Dermatology, Beijing Youan Hospital, Capital Medical University, Beijing, China

**Keywords:** syphilis, treatment failure, cerebrospinal fluid, serum titer, neurosyphilis

## Abstract

**Background:**

In recent years, the incidence of syphilis has increased year by year. Our study is to explore the risk factors for the development of neurosyphilis in patients who failed syphilis treatment.

**Methods:**

A total number of 165 patients with complete medical records and who agreed to undergo lumbar puncture were divided into 47 neurosyphilis cases and 118 non-neurosyphilis cases according to the diagnostic criteria of neurosyphilis, and the differences in clinical characteristics and laboratory features between the two groups were analyzed. Significant variables were entered into multivariable logistic regression models.

**Results:**

(1) There were statistical differences (*p* < 0.05) between the neurosyphilis (NS) group and the non-neurosyphilis (NNS) group in terms of the higher proportion of male and serum rapid plasma reagin (RPR) > 1:32 and the elevated cerebrospinal fluid white blood cell (CSF WBC) and CSF protein in the neurosyphilis group compared with the non-neurosyphilis group. (2) Male gender, serum RPR titers >1:32 at lumbar puncture, CSF WBC >8 × 10^6^/L were significantly associated with neurosyphilis.

**Conclusion:**

For patients who have failed syphilis treatment, lumbar puncture should be performed to exclude neurosyphilis, to enable early diagnosis and treatment, and to prevent irreversible damage of neurosyphilis, especially if the patient is male and has a serum RPR>1:32 and elevated CSF WBC at lumbar puncture, which are risk factors for neurosyphilis.

## Introduction

Syphilis is a sexually transmitted disease caused by Treponema pallidum, which may cause damage to multiple important organs, which seriously endangers human health. The incidence of syphilis has been increasing year by year in recent years, especially after standard anti-syphilitic treatment, some studies reported the treatment failure in patients with syphilis (1, 2), making the treatment of syphilis difficult. Neurosyphilis is often irreversible once severe neurological symptoms have developed, even with therapeutic measures. Thus, it is very important to pay attention to the syphilis treatment failure patients, and block its progression into neurosyphilis. There is little analysis of the factors associated with neurosyphilis in patients who have failed treatment in HIV-negative patients, so this study was conducted to investigate the risk factors for the development of neurosyphilis in patients with treatment failure in a clinical analysis of patients with syphilis.

## Methods

### Study Design

The medical records of syphilis patients who visited our hospital, from January 2014 to December 2020 was retrieved, 165 patients who met the diagnosis of treatment failure with complete medical records and consented to undergo lumbar puncture were screened. Clinical data were collected, including age, gender, marital status, mode of transmission, stage of syphilis, previous treatment (treatment method and times of treatment), the time between diagnosis of syphilis and lumbar puncture, presence of neurosyphilis symptoms (headache, visual symptoms, hearing loss, motor dysfunction, memory loss, etc.), and serum RPR titres, Cerebrospinal fluid (CSF) white blood cells (WBC), CSF protein, CSF rapid plasma reagin (RPR), CSF treponema pallidum particle agglutination (TPPA) at lumbar puncture.

The enrolled patients were divided into neurosyphilis (NS) group and non-neurosyphilis (NNS) group according to the laboratory criteria for neurosyphilis. Ethical approval was obtained from the Institutional Review Board of Beijing Youan Hospital. All patients who participated in the trial signed informed consent.

### Inclusion Criteria and Exclusion Criteria

#### Inclusion Criteria

(1) Met the diagnostic criteria for syphilis, with positive TPPA and RPR. (2) Met criteria for syphilis treatment failure (3, 4). Treatment failure was defined as less than four-fold decrease in the serum RPR titer at or beyond 12 months post-treatment in case of early syphilis, and, at or beyond 24 months in case of late syphilis.

#### Exclusion Criteria

(1) Patients with other meningitis and encephalitis; intracranial tumors; spinal cord lesions caused by other reasons; abnormal cerebrospinal fluid due to trauma, inflammation, immune diseases, etc. (2) HIV-positive patients.

**Figure 1:**
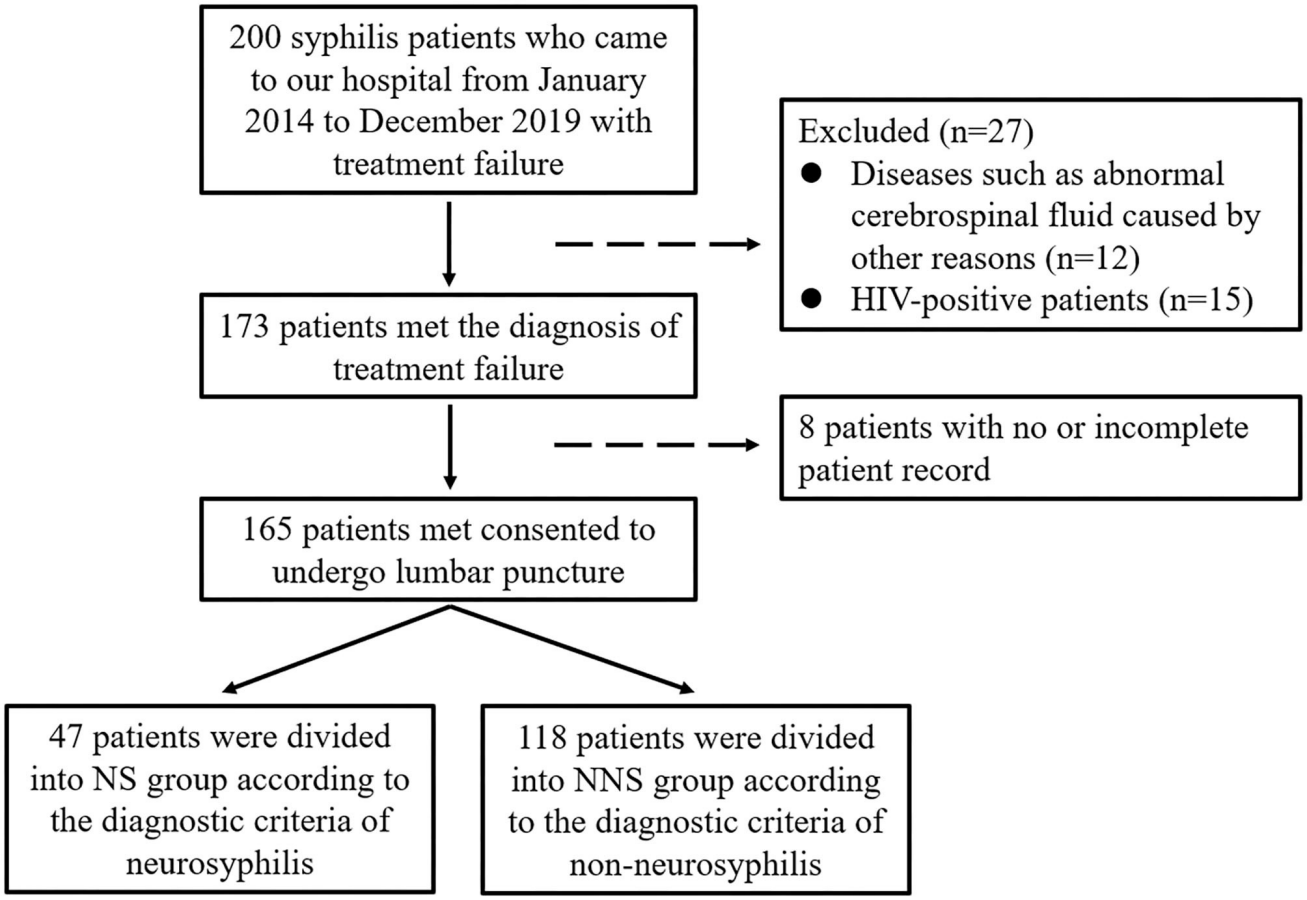
Study flowchart.

The diagnosis of neurosyphilis remains challenging as there are no gold standard tests. The diagnostic criteria for neurosyphilis in our study (5): (1) cerebrospinal fluid examination: white blood cell count > 8 × 10^6^/L, and/or a CSF protein concentration > 450 mg/L (in our study, the normal value of white blood cells in the reagents used in our hospital is ≤ 8 × 10^6^/L, and the normal standard value of protein is ≤ 450 mg/L, so the abnormal standard is set as this), additionally, (2) all patients with NS tested positive for CSF RPR or TPPA in the absence of other known causes of these abnormalities, and without blood contamination. Meeting the above can be diagnosed as neurosyphilis. Procedure of study flowchart can be shown in Figure 1.

### Statistical Analysis

We used SPSS 20.0 to perform statistical analyses. Variables that conform to a normal distribution were described using the Mean and Standard Deviation Model ($\bar{x}$ ± SD), while variables that conform to an abnormal distribution were described by the median and interquartile range (IQR). The Mann-Whitney U test was used to compare continuous variables and the chi-square test was used to compare categorical variables. Significant variables of the Mann-Whitney U test and chi-square test (*p* < 0.05) were entered into multivariable logistic regression models. *p* less than 0.05 were considered to be statistically significant.

## Results

### Characteristics of the Study Population

There were 41 male patients (34.75%) in the NNS group and 30 male patients (63.83%) in the NS group, with a statistical difference between the two groups (*p* = 0.001). Early syphilis included primary, secondary, and early latent syphilis. Late syphilis included late latent and syphilis of unknown duration. There was no primary syphilis because most patients did not recall early syphilis symptoms and syphilis was not detected until physical examination or surgery. There were more patients with late latent syphilis or unknown stages of syphilis, and there was no statistical difference between the two groups with different stages of syphilis. There were 9 cases (7.63%) with neurological symptoms in the NNS group and 8 cases (17.02%) in the NS group, with no statistical difference between the two groups. Patients with early syphilis received 2 weekly intramuscular (IM) injections of 2.4 million units of benzathine penicillin G (BPG), patients with late syphilis and unknown duration received 3 weekly IM injections of 2.4 million units BPG. Penicillin allergic patients were given doxycycline 0.1 g bid orally for 15 days for early syphilis and 30 days for late syphilis. Some penicillin-allergic patients were treated with ceftriaxone 2.0 g for 10–14 days. There were no statistical differences in treatment modality and whether treatment was repeated in these two groups. There were 33 patients (27.97%) in the NNS group and 13 patients (27.66%) in the NS group who showed a decrease in RPR titer after anti-syphilitic treatment, but then RPR titer increased (< four-fold), resulting in a < four-fold decrease in RPR and treatment failure. The RPR of these patients has slightly fluctuation, differing from that of patients (72.03 and 72.34%) whose RPR decreases consistently after treatment. There was no statistically significant difference in the proportion of RPR variation between the NS and NNS groups. CSF WBC and CSF protein at baseline were not statistically different in the two groups. In the NS group, 45 cases showed elevated CSF WBC and 7 cases showed elevated CSF protein. In the NNS group, 2 cases showed elevated CSF WBC and no case showed elevated CSF protein. The proportion of patients with serum RPR > 1:32 at lumbar puncture was 38.30% in the NS group and 8.47% in the NNS group. The proportion of patients with serum RPR > 1:32 at lumbar puncture, CSF protein, and CSF WBC values were higher in the NS group than in the NNS group (*p* < 0.05). Other detailed information on sample characteristics is included in Table 1.

**Table 1:** Demographic and clinical characteristics of the included patients.

	**Non-NS** **group (118) Median (IQR)/N (%)**	**NS group (47)** **Median (IQR)/N (%)**	***p*** **value**
Age, years	36 (28.25, 51.75)	41 (32, 54)	0.135
Gender			
Male	41 (34.75%)	30 (63.83%)	0.001
Female	77 (65.35%)	17 (36.17%)	
Marital status			
Single	47 (39.83%)	19 (40.43%)	0.944
Married	71 (60.17%)	28 (59.57%)	
Route of infection			
Same sex	14 (11.87%)	5 (10.64%)	0.955
Heterosexual	70 (59.32%)	29 (61.70%)	0.793
Unspecified	34 (28.81%)	13 (27.66%)	0.839
Time from diagnosis of syphilis to lumbar puncture (years)	2 (1, 3)	1 (1, 2)	0.556
Neurological symptoms			
Symptomatic	9 (7.63%)	8 (17.02%)	0.073
	2 headache	1 headache	
	2 Dizziness	2 Memory loss	
	3 Blurred vision	2 Tinnitus	
	1 lightning-like pain in the lower limb	1 pain in the left lower limb	
	1 Impatience, babbling	1 Impatience	
Asymptomatic	109 (92.37%)	39 (82.98%)	
Syphilis stage			
Primary	0	0	0.587
Secondary	6 (5.09%)	4 (8.51%)	NS
Early latent syphilis	14 (11.86%)	7 (14.89%)	1.000
Late latent syphilis or unknown stages	98 (83.05%)	36 (76.60%)	0.538
RPR variation			
RPR slightly fluctuation	33 (27.97 %)	13 (27.66%)	0.968
RPR continuous decreased	85 (72.03%)	34 (72.34%)	
Treatment			
Benzathine penicillin G	91 (77.12%)	34 (72.34%)	0.278
Doxycycline	6 (5.08%)	6 (12.77%)	0.097
Ceftriaxone	5 (4.24%)	3 (6.38%)	0.927
Unknown	16 (13.56%)	4 (8.51%)	0.629
Whether to repeat treatment			
Yes	57 (48.31%)	17 (36.17%)	0.157
No	61 (51.69%)	30 (63.83%)	
Serum RPR titer at lumbar puncture			
≤ 1:32	108 (91.53%)	29 (61.70%)	0.001
>1:32	10 (8.47%)	18 (38.30%)	
CSF protein (mg/L)	163.5 (125, 236.5)	293 (197, 417)	<0.001
CSF WBC (×10^6^/L)	3 (2, 4)	12 (9, 28)	<0.001

*NS, not significant*.

### Factors Associated With Neurosyphilis

The logistic regression analysis included gender, serum RPR titer at lumbar puncture, CSF WBC, CSF protein. The regression result showed that male patients (OR 2.131, CI 1.007–6.128, *p* = 0.046) were more prone to neurosyphilis. The serum RPR titer at lumbar puncture >1:32 (OR 3.143, CI 1.325–10.946, *p* = 0.006) and CSF WBC >8 × 10^6^/L (OR 1.053, CI 1.016–1.091, *p* = 0.005) were significantly associated with neurosyphilis. However, there was no correlation between CSF protein and neurosyphilis, and there was no statistical significance. The characteristics of the 165 patients were shown in Table 2.

**Table 2:** Logistics regression analysis of factors related to neurosyphilis diagnosis.

	**OR**	**95% CI**	***p***
Gender (male)	2.131	(1.007, 6.128)	0.046
Serum RPR titer at lumbar puncture (>1:32)	3.143	(1.325, 10.946)	0.006
CSF WBC (×10^6^/L)	1.053	(1.016, 1.091)	0.005
CSF protein (mg/L)	1.003	(0.999, 1.006)	0.114

## Discussions

The number of syphilis cases worldwide has been gradually increasing in recent years (6), and the incidence of syphilis in China is also on a linear increase, accompanied by a high rate of treatment failure, and the causes of treatment failure are still unclear. Scholars concluded that the reasons for the failure of syphilis treatment are as follows: (1) Central nervous system intervention (2), (2) Treponema pallidum (*T. pallidum*) genotype is consistent with macrolide resistance and has appeared in some patients with treatment failure (7, 8), and (3) Patients with treatment failure have abnormal immune function. Some scholars found that such cytokines IL-6, IL-10, TNF-α are involved in the pathogenesis and development of syphilis, with the progression of the disease cellular immune function being somewhat suppressed (9).

Syphilis at any stage can cause damage to the central nervous system. The studies of the incidence of neurosyphilis in patients with treatment failure are limited. A previous study (9) investigated 260 syphilis patients due to treatment failure, confirmed neurosyphilis in 41 patients, with a prevalence of 15.77% (41/260). The incidence of neurosyphilis in patients with treatment failure in our study was 28.48% (47/165), the difference in results of the two studies may be attributed to the small sample size of our study. Only 8 patients in the NS group had neurosyphilis symptoms (17.02%), while the rest had asymptomatic neurosyphilis (82.98%). Therefore, clinically, patients with syphilis who have failed treatment should be highly alert to the possibility of neurosyphilis. Even if the patient has no neurological symptoms, a cerebrospinal fluid examination should be performed to exclude neurosyphilis, so that early diagnosis and treatment can be achieved and further irreversible damage caused by neurosyphilis can be prevented. The 2020 European guideline on the management of syphilis also states (10) that lumbar puncture should be performed to exclude the possibility of neurosyphilis in patients who have failed treatment, even if there are no neurological symptoms. The results of logistic regression in our study showed that male gender and serum RPR >1:32 at lumbar puncture may be risk factors for neurosyphilis. Xiao et al. (11) investigated that symptomatic neurosyphilis was independently associated with male gender, serum RPR titer ≥1:4, serum TPPA titer ≥1:2,560, and these factors increased the likelihood of symptomatic neurosyphilis. In our study, it was suggested that males are more likely to develop neurosyphilis, suggesting that male patients are still the main risk population of neurosyphilis, which is consistent with some previous studies (12–14). At present, it is not clear why men are more likely to develop neurosyphilis, and relevant studies are few, we speculated the reason for gender-related differences could be that unhealthy sexual behavior was more common among men. In addition, gender risk analysis of stroke found that estrogen has a protective effect on the nervous system (15). Whether this mechanism can explain the higher incidence of neurosyphilis in male patients needs further study. Further research is needed to explore the potential factors driving the apparent gender difference. Therefore, in clinical practice, men with treatment failure should be encouraged to undergo a lumbar puncture to avoid missing the diagnosis of neurosyphilis.

The possibility of treatment failure in neurosyphilis is currently present regardless of the treatment used (16). In our study, syphilis stage, treatment modality (penicillin, doxycycline, ceftriaxone, etc.), and whether treatment was repeated were not found to be associated with the development of neurosyphilis in patients who failed treatment. Ceftriaxone crosses the blood-brain barrier and can be used as an alternative treatment for neurosyphilis. The few people treated with ceftriaxone in our study, 6 cases in the NS group and 3 cases in the NNS group, may account for the lack of statistically significant differences between the two groups.

In the previous study, neurosyphilis patients were more likely to have neurological symptoms than non-neurosyphilis patients in both HIV-negative and HIV-positive patients (17). There was no significant difference in the incidence of neurological symptoms in our study, which may be related to the small sample size in our study, or the fact that patients with severe neurological symptoms did not visit our hospital (there was no neurology department in our hospital). Previous studies concerned with risk factors of neurosyphilis in syphilis individuals found that neurosyphilis was more prevalent in higher serum RPR titers (11), and in our study serum RPR titers were higher in NS patients than in NNS patients, especially in patients with serum RPR > 1:32 was a risk factor for the development of neurosyphilis. Therefore, it is suggested that patients who have failed treatment for syphilis with a serum RPR > 1:32, even if they do not have symptoms related to neurosyphilis, should be taken seriously enough to have a cerebrospinal fluid examination to exclude neurosyphilis to avoid missing the diagnosis of neurosyphilis. Although the CSF protein concentration in the neurosyphilis group was higher than in the non-neurosyphilis group, the difference was not statistically significant. The 2020 European guideline on the management of syphilis (10) also states that CSF protein in patients with neurosyphilis may be normal. In contrast, 45 of the 47 cases (95.74%) in the neurosyphilis group had an elevated CSF WBC count, with a statistically significant difference (*p* = 0.005). Thus, an abnormal CSF WBC count had a high diagnostic value for neurosyphilis.

In our study, the RPR of some patients (27.97 and 27.66%) have slightly fluctuation, differing from that of patients (72.03 and 72.34%) whose RPR decreases consistently after treatment. There was no statistically significant difference in the proportion of patients in this group between the NS and NNS groups. The reason for this fluctuation was unclear as they denied new sexual contact and had no new symptoms of hard chancre or syphilid, RPR increased less than four-fold and reinfection was excluded, considering the possibility of laboratory error. It is suggested that patients with syphilis should be monitored regularly for RPR after treatment, and even if treatment is effective at the start, and they should be reminded to follow up regularly for RPR, to monitor closely if there is a fluctuation in RPR, and to perform lumbar puncture if necessary to exclude neurosyphilis.

The limitations of our study were as follows: first, this was a retrospective study, and the research cycle was relatively long, which lead to the omission of patient information collection, which may result in bias. Second, the data for this study were collected from patients who underwent lumbar puncture examination, which may lead to potential bias in patient selection. Since lumbar puncture is an invasive treatment, patients who agreed to undergo lumbar puncture for treatment failure were included in our study, and patients who did not agree to lumbar puncture were not included in the study, which probably resulted in a bias in the results. Third, there was a possibility that some cases were misclassified because of the lack of a gold standard for the diagnosis of neurosyphilis. Some studies (18–21) have reported that the CSF TPPA titers and the level of CSF Chemokine ligand 13 (CXCL13) may be factors for the diagnosis of neurosyphilis. However, these factors are not evaluated in our hospital, so the diagnostic results may be affected. This requires further comprehensive research.

## Conclusion

Male gender, serum RPR > 1:32, and elevated CSF WBC are risk factors for neurosyphilis. For patients who have failed syphilis treatment, the cerebrospinal fluid examination should be performed to exclude neurosyphilis even if the patient is asymptomatic, so that early diagnosis and treatment can prevent irreversible damage to neurosyphilis. In particular, patients should be alerted to the development of neurosyphilis if they are male, have a serum RPR > 1:32 and have an elevated CSF WBC.

## Data Availability Statement

The original contributions presented in the study are included in the article/supplementary material, further inquiries can be directed to the corresponding author/s.

## Ethics Statement

The studies involving human participants were reviewed and approved by Beijing Youan Hospital, Capital Medical University. The patients/participants provided their written informed consent to participate in this study. Written informed consent was obtained from the individual(s) for the publication of any potentially identifiable images or data included in this article.

## Author Contributions

WC conceived the study. JY and WW participated in its design and coordination. WC, YG, and WZ helped to draft the manuscript. All authors read and approved the final manuscript.

## Conflict of Interest

The authors declare that the research was conducted in the absence of any commercial or financial relationships that could be construed as a potential conflict of interest.

## Publisher's Note

All claims expressed in this article are solely those of the authors and do not necessarily represent those of their affiliated organizations, or those of the publisher, the editors and the reviewers. Any product that may be evaluated in this article, or claim that may be made by its manufacturer, is not guaranteed or endorsed by the publisher.

## References

[1] Luo ZZ Zhu L Ding Y Yuan J Li W Wu QH. Factors associated with syphilis treatment failure and reinfection: a longitudinal cohort study in Shenzhen, China. BMC Infect Dis. (2017) 17:620. 10.1186/s12879-017-2715-z 28903736 PMC5598031

[2] Zhang Y Wang J Wei YN Liu HL Wu CL Qu B. CXCL13 concentration in latent syphilis patients with treatment failure. Open Med. (2020) 15:635–43. 10.1515/med-2020-0204 33336020 PMC7712197

[3] Workowski KA Berman S Centers for Disease Control and Prevention (CDC). Sexually transmitted diseases treatment guidelines, 2010. MMWR Recomm Rep. (2010) 59:1–110. 21160459

[4] Kaplan JE Benson C Holmes KK Brooks JT Pau A Masur H. Guidelines for prevention and treatment of opportunistic infections in HIV-infected adults and adolescents. MMWR Recomm Rep. (2009) 58(RR-4):1–207; quiz CE1-4. 19357635

[5] National Center for STD Control Chinese Center for Disease Control and Prevention Venereology Group Chinese Chinese Society of Dermatology Subcommittee Subcommittee on Venereology China Dermatologist Association. Guidelines for diagnosis and treatment of syphilis, gonorrhea and genital Chlamydia trachomatis infection. Chin J Dermatol. (2020) 53:168–79. 10.35541/cjd.20190808

[6] Chen GH Cao Y Yao Y Li M Tang WM Li JJ. Syphilis incidence among men who have sex with men in China: results from a meta-analysis. Int J STD AIDS. (2017) 28:170–8. 10.1177/0956462416638224 26992411 PMC5026914

[7] Stamm LV Bergen HL. A point mutation associated with bacterial macrolide resistance is present in both 23S rRNA genes of an erythromycin-resistant *Treponema pallidum* clinical isolate. Antimicrob Agents Chemother. (2000) 44:806–7. 10.1128/AAC.44.3.806-807.2000 10755994 PMC89774

[8] Zhou PG Li K Lu HK Qian YH Gu X Gong WM. Azithromycin treatment failure among primary and secondary syphilis patients in Shangha. Sex Transm Dis. (2010) 37:726–9. 10.1097/OLQ.0b013e3181e2c753 20644500 PMC3114640

[9] Zhang Y Wu CL Qu B Qiu XM Liang LZ Yan YX. Analysis of detection results of blood and cerebrospinal fluid interleukin-6, 10 and tumor necrosis factor-α in 260 patients with syphilis treatment failure. Zhejiang J Integr Trad Chin Western Med. (2016) 26:341–4. 10.3969/j.issn.1005-4561.2016.04.014

[10] Janier M Unemo M Dupin N Tiplica GS Potočnik M Patel R. 2020 European guideline on the management of syphilis. J Eur Acad Dermatol Venereol. (2021) 35:574–88. 10.1111/jdv.16946 33094521

[11] Xiao Y Tong ML Liu LL Lin LR Chen MJ Zhang HL. Novel predictors of neurosyphilis among HIV-negative syphilis patients with neurological symptoms: an observational study. BMC Infect Dis. (2017) 17:310. 10.1186/s12879-017-2339-3 28446129 PMC5406894

[12] Wang HY Chen QH Lu XO Cao XY Yan J. Analysis of clinical data of 36 cases with neurosyphilis. China J Leprosy Skin Dis. (2020) 36:275–7, 281. 10.12144/zgmfskin202005275

[13] Chen HZ Xie J Li SS Chen NN. Retrospective analysis of clinical data of 20 patients with neurosyphilis. Dermatol Venereol. (2021) 4:497–8. 10.3969/j.issn.1002-1310.2021.04.016

[14] Chen Y Gu HY Zhang L Wang LH Li XW. The analysis of the clinical and epidemiological features of 117 cases of neurosyphilis. Chin J AIDS STD. (2015) 21:879–883. 10.13419/j.cnki.aids.2015.10.15

[15] Broughton BRS Brait VH Kim HA Lee S Chu HX Gardiner-Mann CV. Sex-dependent effects of G protein-coupled estrogen receptor activity on outcome after ischemic stroke. Stroke. (2014) 45:835–41. 10.1161/STROKEAHA.113.001499 24457292

[16] Sena AC Wolff M Martin DH Behets F Damme KV Leone P. Predictors of serological cure and serofast state after treatment in HIV-negative persons with early syphilis. Clin Infect Dis. (2011) 53:1092–9. 10.1093/cid/cir671 21998287 PMC3205200

[17] Lu Y Ke W Yang L Wang Z Lv P Gu J. Clinical prediction and diagnosis of neurosyphilis in HIV-negative patients: a case-control study. BMC Infect Dis. (2019) 9:1017. 10.1186/s12879-019-4582-2 31791265 PMC6886180

[18] Yan YX Wang J Qu B Zhang Y Wei YN Liu HL. CXCL13 and TH1/Th2 cytokines in the serum and cerebrospinal fluid of neurosyphilis patients. Medicine. (2017) 96:e8850. 10.1097/MD.0000000000008850 29381995 PMC5708994

[19] Zeng YL Lin YQ Zhang NN Zou CN Zhang HL Peng F. CXCL13 chemokine as a promising biomarker to diagnose neurosyphilis in HIV-negative patients. Springerplus. (2016) 5:743. 10.1186/s40064-016-2462-4 27376011 PMC4909691

[20] Gudowska-Sawczuk M Mroczko B. Chemokine ligand 13 (CXCL13) in neuroborreliosis and neurosyphilis as selected spirochetal neurological diseases: a review of its diagnostic significance. Int J Mol Sci. (2020) 21:2927. 10.3390/ijms21082927 32331231 PMC7216086

[21] Shiva F Goldmeier D Lane P Ethiopia H Winston A. Cerebrospinal fluid TPPA titres in the diagnosis of neurosyphilis. Sex Transm Infect. (2020) 96:389–90. 10.1136/sextrans-2019-054198 31959701

